# Hematopoietic Cell Transplantation for Sickle Cell Disease

**DOI:** 10.3389/fped.2020.551170

**Published:** 2021-01-05

**Authors:** Lakshmanan Krishnamurti

**Affiliations:** Aflac Cancer and Blood Disorders Center, Children's Healthcare of Atlanta, Emory University, Atlanta, GA, United States

**Keywords:** transplantation, hematopoietic stem cell, sickle cell disease, sickle cell anemia, gene editing, clinical trial, curative treatment

## Abstract

Sickle cell disease (SCD) is a severe autosomal recessively inherited disorder of the red blood cell characterized by erythrocyte deformation caused by the polymerization of the abnormal hemoglobin, which leads to erythrocyte deformation and triggers downstream pathological changes. These include abnormal rheology, vaso-occlusion, ischemic tissue damage, and hemolysis-associated endothelial dysfunction. These acute and chronic physiologic disturbances contribute to morbidity, organ dysfunction, and diminished survival. Hematopoietic cell transplantation (HCT) from HLA-matched or unrelated donors or haploidentical related donors or genetically modified autologous hematopoietic progenitor cells is performed with the intent of cure or long-term amelioration of disease manifestations. Excellent outcomes have been observed following HLA-identical matched related donor HCT. The majority of SCD patients do not have an available HLA-identical sibling donor. Increasingly, however, they have the option of undergoing HCT from unrelated HLA matched or related haploidentical donors. The preliminary results of transplantation of autologous hematopoietic progenitor cells genetically modified by adding a non-sickling gene or by genomic editing to increase expression of fetal hemoglobin are encouraging. These approaches are being evaluated in early-phase clinical trials. In performing HCT in patients with SCD, careful consideration must be given to patient and donor selection, conditioning and graft-vs.-host disease regimen, and pre-HCT evaluation and management during and after HCT. Sociodemographic factors may also impact awareness of and access to HCT. Further, there is a substantial decisional dilemma in HCT with complex tradeoffs between the possibility of amelioration of disease manifestations and early or late complications of HCT. The performance of HCT for SCD requires careful multidisciplinary collaboration and shared decision making between the physician and informed patients and caregivers.

## Introduction

Sickle cell disease (SCD) is an autosomal recessively inherited disorder of the red blood cell (RBC) (1) characterized by the polymerization of the abnormal hemoglobin. Polymerization of the hemoglobin leads to erythrocyte deformation triggering downstream pathological changes, including abnormal rheology, vaso-occlusion mediated by abnormal adhesivity, ischemic tissue damage, and hemolysis-associated endothelial function. These acute and chronic physiologic disturbances contribute to the morbidity, organ dysfunction, impairment of health-related quality of life (QoL) (2), and diminished survival (3, 4). SCD is a major public health problem in the United States affecting more than 100,000 individuals (5), predominantly African Americans, and is associated with substantial healthcare costs. Hydroxyurea, l-glutamine (6), voxelotor (7), and crizanlizumab (8) offer the possibility of the modification and amelioration of the disorder. Patients have to take these medications indefinitely, and access to care, health disparities, and other sociodemographic factors may influence uptake, usage, and effectiveness of long-term disease modification therapy. Hematopoietic cell transplantation (HCT) results in excellent outcomes in excellent overall survival (OS) and SCD-free survival (DFS) following matched related donor (MRD) HCT (9–13). There is increasing application of HCT from matched siblings, or from alternate donors (14–17) using myeloablative, reduced intensity, or non-myeloablative (NMA) conditioning regimens (15, 18–21). More than 65% of HCTs for SCD procedures reported from 2008 to 2017 were performed after 2013 (22). While the majority of HCTs performed have been from an MRD, recently, there is increasing use of alternative donor HCT (22). There are more than 100,000 individuals in the United States with SCD (5). There are many millions of patients worldwide, with more than 300,000 newborns with SCD born each year (23, 24). Therefore, we anticipate that increasing numbers of patients worldwide will consider the option of HCT for SCD. Further, the transplantation of autologous hematopoietic cells modified by gene addition or genomic editing offers the possibility of expanding the application of HCT for SCD (25–27).

## Indications for HCT

HCT for SCD has been typically reserved for patients with severe complications of SCD, such as stroke or who were considered to be at risk of long-term disease-related complications (Table 1). In the initial clinical trials of HCT for SCD, the most common indication for HCT was stroke in 57% of patients and frequent vaso-occlusive pain crises (VOCs) in 23% of patients (9). In contrast, in recent studies, recurrent episodes of pain exacerbation requiring healthcare utilization have been reported as the most frequent indication for HCT (13, 18, 28). That patients are increasingly seeking HCT because of the impact of recurrent acute and possibly chronic pain on their QoL suggests that HCT is moving from a lifesaving treatment to one intended to improve the QoL. The number of hospitalizations or emergency department (ED) visits for SCD-associated pain is a poor surrogate measure of the total burden of pain. Patients manage most of their pain at home; hence, a hospital or ED visit represents a small fraction of the patients' pain experience (29). The duration of daily pain and the presence of disability provide a better measure of the impact of pain on the lives of individuals with SCD and is beginning to be used as an eligibility criterion for HCT for SCD (30, 31).

**Table 1:** Disease severity criteria for consideration of HCT for SCD adapted from Walters et al. (9).

Stroke or CNS event lasting >24 h
ACS with recurrent hospitalizations or previous exchange transfusion
Recurrent VOC (≥2/year for several years) or recurrent priapism
Impaired neuropsychological function and abnormal brain MRI
Stage I or II sickle lung disease
Sickle nephropathy (moderate or severe proteinuria or GFR 30–50% of predicted)
Bilateral proliferative retinopathy and major visual impairment in at least one eye
Osteonecrosis of multiple joints
Red cell alloimmunization (≥2 antibodies) during long-term transfusion therapy

No randomized controlled trials have compared HCT with non-transplant treatment therapies such as hydroxyurea; however, favorable long-term survival has been reported on hydroxyurea. The efficacy of other disease-modifying therapies must be weighed against the fact that they need to be continued indefinitely and that morbidity, QoL, and the risk of premature death worsen with age in SCD patients. An ongoing trial (*NCT02766465*, BMT CTN 1503) compares the outcomes of patients with severe disease and an available donor who undergo HCT and those who do not have a donor and continue observation on standard clinical care.

An international expert panel recommended the consideration of HCT in young patients preferably at preschool age with symptomatic SCD who have an available HLA-identical sibling donor (32). The panel recommended using bone marrow and umbilical cord blood (UCB) from HLA-identical sibling donors as the preferred stem cell sources. They also suggested that HCT from an alternate donor source be restricted to those with severe disease, preferably in a clinical trial and at a center experienced in the performance of the procedure.

## Decision Making for HCT for SCD

### Recipient Considerations

Recipient factors in the consideration or decision making for HCT and used as eligibility criteria in clinical trials have typically included the severity of the phenotype, including QoL, adequacy of organ function, response to current disease-modifying therapies, and the imminence of future complications (Table 1). In initial reports of HCT, patients had undergone the procedure most commonly because of a history of stroke (9), although more recently, recurrent VOC requiring medical care is reported as the most common indication for HCT (12).

The best OS and event-free survival (EFS) are reported in children younger than 5 years with an increased risk of complications with each year of increasing age (12, 14, 33). SCD-related organ damage progresses with age. SCD-related damage to the kidneys (34) and to the spleen (35) commences in infancy. Silent cerebral infarction is observed in nearly one-quarter of patients under 6 years of age (36). Silent cerebral infarcts are associated with cognitive deficits and poor school performance (37). High rates of OS and EFS in adult patients undergoing MRD HCT (13, 18) have expanded the applicability of HCT in this age group. Thus, HCT from an HLA-identical sibling donor may be considered for young children without the severe disease phenotype because of excellent outcomes and the potential for the patient to have a normal childhood and avoid disease progression and complications of SCD in later life. However, these benefits must be carefully weighed against the risk of late effects such as infertility and subsequent malignancy.

### Donor Considerations

The availability and use of an HLA matched donor are the primary donor factor that impacts outcomes. Stem cell source and donor age are important secondary considerations. The majority of sibling donors have sickle cell trait (HbAS) (11), but a heterozygous carrier state in the donor does not appear to impact outcomes. HCT using UCB from an HLA-identical sibling is associated with excellent outcomes. An acceptable total nucleated cell (TNC) dose for UCB is at least 3 × 10^7^ TNC/kg recipient weight (38). However, in one case series in which a median dose of 3.9 × 10^7^ TNC/kg (range, 1.5–14 × 10^7^ TNC/kg) was administered, no impact was observed of cell dose administered on engraftment or DFS (39). If the cell dose is low with TNC 1 × 10^7^/kg to 3 × 10^7^/kg, a combination of UCB and bone marrow may be infused (10). Donor weight exceeding 10 kg and age exceeding 1 year are generally considered safe for collecting an adequate cell dose. If more than one HLA-identical–related donor is available, additional factors to consider in donor selection include donor size/age, ABO typing, and cytomegalovirus (CMV) serostatus. Donor age may impact the risk of chronic graft-vs.-host disease (GVHD) risk in malignant disease (40, 41) and may similarly impact HCT recipients with SCD. ABO major or minor mismatch may add to the risk of graft stem cell loss (42). Major ABO mismatch should be avoided as it may be associated with delayed RBC engraftment (43), decreased OS (44), and pure red cell aplasia (45). Donor and recipient should be matched for CMV serostatus whenever possible to minimize risks of CMV disease.

### Decision-Making by Patient, Families, and Caregivers

HCT for SCD poses a decisional dilemma involving tradeoffs between the possibility of cure, amelioration of symptoms on the one hand and the risk of acute toxicities and late effects on the other hand (46). Advances in care have greatly improved outcomes of SCD, but there is a lack of data comparing outcomes of HCT with that of standard clinical care. HCT has curative intent but is associated with treatment-related morbidity and mortality risk. The perceived severity of disease and availability of a suitable donor influence the decision-making by SCD patients, their caregivers (46), and their physicians (47). The decision is also influenced by the availability of family support, resources, and BMT-related education and awareness (46). A quarter of SCD patients and caregivers are unwilling to accept *any* risk of GVHD or mortality (48). Seventy-two percent of parents of a child with SCD were willing to accept a ≥5% risk of mortality, whereas 57% are willing to accept a risk of ≥10% of GVHD (48). Interestingly, following a successful HCT, SCD patients and caregivers do not report decisional regret (46). In that study, even the few patients who were currently dealing with chronic GVHD indicated that although they are dealing with frequent clinic visits and receiving treatments, they were glad to be rid of SCD and its complications.

### The Physician Perspective on Decision Making

Physicians may be influenced by their past experiences, outcomes of previous patients undergoing HCT, their perception of the severity of disease manifestation in the individual, and a variety of patient and institutional characteristics in the decision to refer a patient for consideration of HCT (47). We performed a qualitative study of physician perceptions and approach to decision making regarding disease-modifying therapies in SCD (47). We identified two different narratives in the physician's approach to patient decision making. In the collaborative approach, the physician co-opts the patient in a joint examination of all available treatment options to jointly arrive at a treatment choice. In the proponent approach, on the other hand, the physician strongly advocates a particular treatment and educating patients/families to convince them to accept that treatment. The ethics of this decision-making have been extensively reviewed (49, 50). As the decision for HCT for SCD is complex, shared decision making about HCT must involve the cooperation of the informed and empowered patient and caregivers, the primary hematologist, and the transplant physician.

## Pre-HCT Recipient Evaluations

Eligibility for HCT is examined based on disease severity, the adequacy of organ function, and for any SCD-related or treatment-related complications that may impact the HCT course or outcome.

Before HCT, patients are evaluated for organ damage. SCD-associated neurologic damage is assessed by brain magnetic resonance imaging (MRI)/magnetic resonance angiography. Patients with severe cerebral vasculopathy or moyamoya disease may be considered for encephaloduroarteriosynangiosis (51). Cerebral blood flow velocity determined by transcranial Doppler velocity (age <16 years) and neurocognitive function may stabilize or improve posttransplant (52) and may be evaluated pre-HCT. Transfusional hemosiderosis and its effect are examined by the quantification of the liver and cardiac iron. The presence and severity of liver fibrosis are evaluated by magnetic resonance or ultrasound elastography and by liver biopsy as needed. As patients are at risk of renal dysfunction (34, 53), glomerular filtration rate (GFR), urine specific gravity, and albumin-to-creatinine ratio are evaluated pre-HCT (54). The splenic function is impaired and hence is evaluated pre-HCT with liver–spleen nuclear medicine scan (35) or RBC pit count. These studies are repeated 1 year post-HCT to determine the recovery of splenic function (55). The presence of donor-directed HLA antibodies in high titers may predict an increased risk of graft rejection. Donor-directed HLA antibodies can be reduced by desensitization strategies (56, 57), and patients with SCD have subsequently successfully undergone haploidentical HCT (56). Patients are evaluated by transfusion medicine specialists to assist in assessing the alloimmunization status or special needs for packed RBCs and formulate a plan for the management of RBC transfusion before conditioning and in the peritransplant period (58).

Patients are typically evaluated by social workers, psychologists, and child life specialists and counseled regarding preparation for the anticipated stressors associated with HCT. Patients are encouraged to consult reproductive endocrinologists and receive counseling regarding their options for clinical or research procedures for fertility preservation.

## HCT Conditioning Regimen

Myeloablative conditioning (MAC) has been used most often in MRD HCT (12). The regimen used in the early transplant series consisted of a myeloablative combination of busulfan and cyclophosphamide (9). More recent reports describe the use of reduced-intensity conditioning (RIC) (10, 12, 14, 15, 59, 60). The most common RIC approach has been the substitution of cyclophosphamide with fludarabine in combination with another agent (12), typically an alkylator such as busulfan or melphalan (10). Reduced-toxicity conditioning regimen (61, 62) has also been reported (13, 63, 64), including reduced doses of busulfan in combination with fludarabine with or without cyclophosphamide (63) or substitution of busulfan with treosulfan and the addition of thiotepa (64). NMA conditioning with TBI 200 cGy and fludarabine (65, 66) was associated with poor long-term engraftment. Recently, NMA strategies combining TBI (300 cGy) with alemtuzumab have been associated with high OS and EFS in adults (18, 67) and children (20, 68).

## GVHD Prophylaxis

T-cell depletion *in vivo*, using antithymocyte globulin (ATG; 70.6%) or alemtuzumab (11.5%), has been used in most patients undergoing MRD HCT (12). Bernaudin et al. demonstrated that the use of ATG was associated with the decrease of graft failure rate from 22.6% to 3% (11). ATG use is, however, uncommon in MRD UCB transplant (39). T-cell depletion *in vivo*, with alemtuzumab, has been administered in MRD HCT (10, 18) and URD UCB transplant (15, 59). T-cell depletion *in vivo*, with posttransplant cyclophosphamide with ATG or alemtuzumab (16, 19, 21, 56, 60), has been used for haploidentical HCT for SCD. The use of *ex vivo* T-cell depletion, with CD34^+^ selection (69), CD3/CD19 depletion (70), or T-cell receptor (TCR) α/β and CD19 depletion (71), has also been reported in haploidentical HCT for SCD. *Ex vivo* TCR α/β and CD19 depletion have been associated with a reduction in risk of GVHD, but may, however, be complicated by delayed immune reconstitution, infection, and graft failure (71, 72). Calcineurin inhibitors (CNIs) are the most commonly used GVHD prophylaxis and may be combined with methotrexate or mycophenolate mofetil (12). Locatelli et al. observed decreased DFS following MRD UCB transplant with MTX (39). MMF is substituted in this setting (15, 59). The addition of abatacept, a selective inhibitor of T-cell costimulation, to the GVHD prophylaxis regimen has the potential to decrease the GVHD and thus improve the safety profile and applicability of HCT to SCD (73).

## Cell Dose Considerations

Cell dose predicts engraftment, with the rate of graft failure decreasing to 5% from 10% when TNC was ≥2.5 × 10^8^/kg (74). In unrelated donor transplantation, increased mortality was predicted by a peripheral blood mononuclear cell dose of TNC <2 × 10^8^/kg or a bone marrow graft nucleated cell dose <5 × 10^8^/kg, respectively (75). In URD UCB, TNC >5 × 10^7^/kg increased engraftment and DFS (76). The generally recommended target cell dose is 4 × 10^8^ to 5 × 10^8^ TNC/kg for bone marrow, and 4 × 10^7^ to 5 × 10^7^ TNC/kg for UCB (prethaw) grafts.

## Engraftment Following Transplant

Factors predicting graft failure include the degree of HLA mismatch, the titers of donor-directed HLA antibodies if present, the intensity of conditioning, and the presence of active infection at the time of engraftment (74).

Lineage-specific chimerism in the lymphoid lineage[CD3] and myeloid lineage[CD15 or CD33], as hemoglobin level, and HbS% are typically used to evaluate the degree of engraftment and donor-derived hematopoiesis. Whole-blood chimerism of 11% to 74% may be associated with stable donor-derived erythropoiesis (77–79). Lineage-specific chimerism may provide additional information, although red cell chimerism assays are still under evaluation in research studies (80, 81). Myeloid chimerism of 20–25% (13, 78, 79) may best predict stable donor-derived erythropoiesis. An HbS >50% suggests the likelihood of impending autologous recovery. In the case of mixed or declining donor chimerism or increasing HbS percentage, more frequent assessments may be necessary. The role of donor lymphocyte infusions in improving donor chimerism is unknown and, in any case, associated with a significant risk of GVHD.

The majority of patients rejecting the allograft reconstitute autologous hematopoiesis (11, 78) even in the case of alternative donor HCT (13–16, 78). If marrow aplasia occurs or if prolonged cytopenia is observed, a salvage HCT may be required urgently (13). In patients with autologous reconstitution, following graft failure, a second HCT may be considered after at least 6 months following the first HCT procedure.

## Prevention and Management of Complications During HCT

SCD patients are uniquely susceptible to certain neurologic, cardiovascular, pulmonary, hepatobiliary, renal, and infection risks in the immediate peri-HCT period and the long term (82).

### Neurologic

Neurologic complications include seizures and hemorrhagic stroke, which were observed in 30% of patients of the initial group of patients who underwent MRD HCT (83). The following risk factors for neurologic complications in HCT for SCD were identified: (i) stroke particularly in patients with a history of prior stroke; (ii) hypertension due to neurologic and renal dysfunction exacerbated by nephrotoxic medications such as CNIs and hypertension due to prednisone; (iii) hemorrhagic stroke with concurrent thrombocytopenia in patients with preexisting cerebral vasculopathy exacerbated by concurrent postchemotherapy thrombocytopenia (11), and (iv) a high incidence of posterior reversible encephalopathy syndrome (PRES) (22–34%) (14, 84), with the risk of PRES in SCD patients (85) exacerbated during HCT (84). Patients who developed PRES following MRD HCT had decreased OS and DFS (84). BMT CTN 0601, a multicenter trial of URD BMT, reported a high incidence of PRES (14). Of note, this study included the use of prednisone through day +28 as part of GVHD prophylaxis.

Thus, precautions instituted to decrease neurologic complications include (i) drug prophylaxis to prevent seizures starting prior to conditioning, especially if busulfan is used and continued for the duration of CNI administration, and (ii) strict control of hypertension. Particular caution must be exercised in the monitoring and management of hypertension when patients with SCD are receiving both prednisone and CNI post-HCT because both drugs are likely to cause hypertension. Blood pressures (BPs) in SCD are lower compared to age-matched peers (86). As such, the BP control regimen should take into account these lower BP parameters, as well as the baseline BP of the patient (iii) magnesium supplementation to prevent hypomagnesemia (87, 88) and (iv) platelet transfusions to maintain platelets >50,000/μL and RBC transfusion to maintain hemoglobin 9–11 g/dL.

### Cardiovascular and Pulmonary

Individuals with SCD run lower BP compared to individuals matched for age, sex, and race (86). BP above the 50th percentile for age may be associated with an increased risk of stroke (86, 89). Thus, the prevention of neurologic complications such as seizures or PRES requires careful monitoring and strict control of BP (9, 83), with a target BP within 10% of the median for age and sex, for SCD patients (86).

A combination of echocardiographic tricuspid regurgitant jet (TRJ) velocity >3.0 m/s, and Brain Natriuretic Peptide BNP >160 pg/mL is a strong predictor of premature mortality in adult SCD patients (90, 91). Successful HCT may be associated with the improvement of TRJ velocity (13).

### Infections

SCD patients undergo autoinfarction of the spleen with impaired splenic dysfunction in most patients by age 3 years (35, 92), thus increasing the risk of pneumococcal sepsis mostly with non-vaccine serotypes (93). Pediatric SCD patients may recover splenic function post-HCT, but older patients and those with extensive chronic GVHD are at risk of poor post-HCT splenic recovery (55). While pneumococcal infections are rare following HCT for SCD (55), deaths from pneumococcal sepsis have been reported post HCT for SCD. As such, pneumococcal prophylaxis during HCT, monitoring of splenic function recovery post-HCT, and timely reimmunization starting with conjugated pneumococcal vaccines 6 months posttransplant (94) is recommended.

### Management of Iron Overload

Patients may have transfusional hemosiderosis pre-HCT and may have received several transfusions of packed red blood cells PRBCS with the HCT. As such, residual iron overload is measured by serum ferritin and MRI at 1 year post-HCT by which time patients are typically off immunosuppression and are transfusion independent. Removal of excess body iron stores must be instituted after HCT and continued with close follow-up monitoring until the resolution of iron overload is demonstrated. Transfusional hemosiderosis may be treated with oral iron chelation, by monthly phlebotomy, or a combination thereof (95–98). The method adopted for reducing iron overload should be dictated by the degree of iron overload and the preferences and circumstances of the individual patient. Individuals with cardiac iron overload must undergo follow-up MRI evaluation of cardiac iron overload post-HCT.

### Renal

Prior to HCT, serum blood urea nitrogen/creatinine and GFR or 24-h creatinine clearance are obtained to determine the adequacy of renal function and are followed yearly for at least 2 years post-HCT. Patients are evaluated for SCD-associated proteinuria pre-HCT and followed for recovery post-HCT (82). Patients who are on prolonged course of CNIs are at high risk of renal dysfunction. Patients with SCD may have prior complement-mediated vascular injury due to their underlying primary hemolytic disease and then again from additional stressors during the transplantation process leading to progressive endothelial injury and end-organ dysfunction (99). This may place them at a greater risk of transplant-associated thrombotic microangiopathy (TA-TMA) and subsequent chronic kidney disease. Patients must be monitored for HCT TA-TMA, and interventions such as control of hypertension, consideration of alternatives to CNIs, and the introduction of eculizumab therapy may be considered prior to overt clinical manifestations (100).

## Outcomes Following HCT

The first HCT for SCD was performed in a child who developed acute myeloid leukemia, thus curing both diseases (101). Since the first multicenter clinical trial (9), HCT for SCD has been rapidly expanding in its applicability. The majority of patients reported are children who have undergone HCT from HLA-identical–related donors (9, 10, 12, 13, 18, 39, 67, 77, 81, 102, 103) Gluckman et al. reported in a joint Eurocord-CIBMTR registry–based study an OS of 92.9% and EFS of 91.4% in 1,000 children who underwent HCT for SCD (12). On multivariate analysis, survival was found to decline with increasing recipient age and was to be higher in patients undergoing HCT after the year 2006. Previously, most patients undergoing HCT received MAC. In more recent series, reduced toxicity/intensity (10, 13, 81) and NMA (18, 20, 67, 103) conditioning regimens have been demonstrated to be safe and effective in ameliorating SCD clinical manifestations (10, 13, 81). Following HCT, the transcranial Doppler velocity returns to normal; there is a stabilization of organ function, stabilization of full-scale IQ, improved health-related QoL (HRQoL), decreased prescriptions of opioid pain medications, and improvement in splenic function (11, 13, 14, 52, 55, 104–107). Adult patients followed in the 2nd year after a successful HCT for SCD, as compared to pre-HCT, and as compared to a group of SCD patients who had not undergone HCT, had significantly lower rates of healthcare utilization and healthcare costs (108).

Following a successful HCT pain eventually resolves in the large majority of SCD patients. Pain, however, may persist for a prolonged period, especially in older patients, those with higher pain burden, with more anxiety, or more use of long-acting opioids prior to HCT (109). While pain intensity may remain unchanged at 1 year in 40% (109), HRQoL may improve, with decreased pain interference and improved physical function (13). Thus, patients with a history of chronic pain with high pain burden and pain medication use pre-HCT and require close follow-up and multidisciplinary rehabilitation post-HCT.

## Predictors of Outcomes

The age at the performance of HCT is the strongest predictor of EFS in HLA-identical sibling donor HCT (12, 33, 104). Brazauskas et al. described a simple risk score to guide patients with SCD and hematologists who are considering HCT as a curative treatment relative to other available contemporary treatments (110). Patients 12 years or younger with MRD were at the lowest risk with a 3-year EFS of 92% (score, 0). Patients 13 years or older with an MRD or those 12 years or younger with an HLA-matched unrelated donor were at intermediate risk (3-year EFS, 87%; score, 1). All other groups, including patients of any age with a haploidentical relative or HLA-mismatched unrelated donor and patients 13 years or older with an HLA-matched unrelated donor, were high risk (3-year EFS, 57%; score, 2 or 3).

## Current Research Gaps

To date, a small proportion of SCD with severe clinical manifestations has undergone HCT. In one survey of SCD programs, only 8% of patients with severe manifestations who would have met eligibility for participation in a trial of HCT for SCD underwent a transplant. The lack of an available MRD is the primary barrier to the consideration of HCT (111). The likelihood of an African American finding a suitable 8 of 8 antigen HLA-matched donor is as low as 19%, with <5% finding a potential matched (6/6) unrelated UCB donor (112, 113). BMT CTN 0601 results revealed an unacceptably high rate of chronic GVHD following URD BMT (13, 14). Conversely, the rates of graft failure following unrelated UCB transplants were found to be unacceptably high (59, 76), but modifications in conditioning may result in improved rates of engraftment (15). The expansion of UCB units *ex vivo* has the potential to improve the applicability of this cell source for HCT for SCD (114). Increasing the cell dose would enable the use of an otherwise suitably HLA-matched UCB, which may have had a cell dose below the threshold of acceptability. HCT from related haploidentical donors can expand the donor pool, thus expanding the applicability of SCD. Early reports suggested that while the safety of the procedure was acceptable, the rates of stable engraftment were low (16). Refinements in conditioning regimen by either the addition of thiotepa or by increasing the dose of TBI have demonstrated improvement in EFS rates (16, 17, 21, 56, 107). Despite improvements in outcomes of MRD and the development of multiple options for alternate donor HCT, concerns for serious short-term complications such as GVHD, as well as long-term risks specifically of infertility or subsequent malignancy, remain a substantial barrier to the acceptability of HCT. Further, awareness of these options remains poor, and access to quality care remains challenging. Health disparities, even in high-resource countries, and the lack of health infrastructure in low-resource countries severely limit the general applicability of HCT for SCD. Thus, there is a need for progress on multiple fronts before HCT can become a standard therapy that is applicable and acceptable to a sizable proportion of patients with SCD.

## Potential Developments in the Field

SCD, the first molecular disease (115), has long been the target for the development of gene therapy for producing long-term amelioration of the disease. Gene addition and genomic editing are two approaches to gene therapy. Gene addition involves the addition of a non-sickling beta-globin gene into the genome of the hematopoietic stem cell. Genomic editing involves the use of molecular techniques to reverse the silencing of the fetal gamma globin gene with a view to increase the expression of non-sickling fetal hemoglobin (27). Early results of the addition of a non-sickling beta-globin gene using a lentiviral vector have been encouraging (116). Multiple studies of genomic editing aimed at fetal hemoglobin induction through targeting BCL11a are ongoing (117–121). Chemotherapy-based conditioning and the resultant bone marrow aplasia, mucositis, and late effects such as infertility remain a barrier to the acceptability of HCT and gene therapy. Non-genotoxic conditioning based on antibody targeting hematopoietic stem cells has the potential to reduce the toxicity of HCT and gene therapy (122–124). The development of novel GVHD prophylaxis such as ruxolitinib, a selective Janus kinase (JAK1 and JAK2) inhibitor (125, 126), and the use of biomarker panels to predict outcomes and direct therapy of GVHD (127) may further improve the safety of HCT and expand its applicability for SCD.

## Summary and Conclusions

Excellent outcomes in MRD HCT, improvements in the conditioning regimen, novel methods of GVHD prophylaxis, and the ability to safely use alternative donors have all contributed to the applicability of HCT for SCD. Encouraging early results in the clinical trials of the feasibility and safety of gene therapy suggest that this approach may further increase the applicability of HCT (116). Hence, the discussion about available curative options should be integrated with the comprehensive care of SCD patients. In young children with symptomatic SCD with an available HLA-identical–related donor, careful consideration should be given to proceeding to HCT, even in the absence of severe SCD-associated comorbidities. HCT from alternative donors is typically undertaken only in patients with severe symptoms, causing or likely to cause organ damage, and undertaken in the context of clinical trials. Patients undergoing these therapies require care and counseling regarding psychosocial aspects, including the importance of adherence to medications, fluid intake, and precautions to prevent infections. The study of long-term outcomes following HCT for SCD (128, 129) through long-term follow-up registries is key to determining long-term efficacy and late effects of HCT (82). The transplantation of autologous gene-modified HPCs is not associated with GVHD and eliminates the need for a matched allogeneic donor. Questions remain, however, regarding the level of non-sickling hemoglobin required for long-term disease amelioration and organ function. Ongoing studies will also address the durability of gene-modified cell engraftment, as well as the long-term risk, if any, of insertional mutagenesis (116, 130, 131). Currently, cost and availability are major barriers to the application of gene therapies (132).

Further research must focus on how to make these treatments generally available and at a reasonable price point. Studies of incremental cost-effectiveness must account for the individual and societal impact of chronic illness, associated utilization of healthcare, and the loss of educational opportunity and suitable employment. There is a need for ongoing clinical and translation research, clinical trials, and long-term follow-up studies, to explore the enormous potential of HCT in the treatment of SCD.

## Author Contributions

This study was conceived, written, and edited by LK.

## Conflict of Interest

The author declares that the research was conducted in the absence of any commercial or financial relationships that could be construed as a potential conflict of interest. The reviewer NS declared a shared affiliation, though no other collaboration, with the author LK.
